# Retrospective Assessment of the Oxygenation Index in Dogs Undergoing Mechanical Ventilation for Primary Pulmonary Parenchymal Disease

**DOI:** 10.1111/vec.70096

**Published:** 2026-02-19

**Authors:** Tereza Stastny, Jiwoong Her, Curtis G. Rheingold, Nick Jordan, Dana J. Caldwell, Rebecka S. Hess, Deborah C. Silverstein, Jeongmin Lee

**Affiliations:** ^1^ University of Pennsylvania School of Veterinary Medicine Philadelphia Pennsylvania USA; ^2^ North Carolina State University College of Veterinary Medicine Raleigh North Carolina USA; ^3^ The Ohio State University College of Veterinary Medicine Columbus Ohio USA; ^4^ Arizona Emergency and Critical Care Center Gilbert Arizona USA

**Keywords:** canine, mechanical ventilation, oxygenation index, parenchymal disease

## Abstract

**Objective:**

To assess the association between the oxygenation index (OI) and survival in mechanically ventilated dogs with primary pulmonary parenchymal disease.

**Design:**

Retrospective, multicenter study.

**Setting:**

Three veterinary medical teaching institutions and one private veterinary referral center.

**Animals:**

Seventy‐nine client‐owned dogs.

**Interventions:**

None.

**Methods:**

OI, calculated as (mean airway pressure × FiO_2_ × 100) / PaO_2_, and the PaO_2_/FiO_2_ (PF) ratio were calculated for dogs undergoing mechanical ventilation for primary pulmonary disease. Median OI was lower in survivors (2.6) than nonsurvivors (6.6; *p* < 0.001), while PF was higher in survivors (317.9 vs. 177.9; *p* < 0.001). OI predicted mortality with an area under the receiver operating characteristic curve of 0.73, sensitivity of 65%, and specificity of 80% at an optimal cutoff of 4.3. Median PF had an area under the receiver operating characteristic curve of 0.72, sensitivity of 70%, and specificity of 73% at an optimal cutoff of 237.8. Each 1‐unit increase in OI was associated with a 35% higher mortality risk (odds ratio: 1.35; 95% confidence interval: 1.14–1.61). Survivors showed greater improvement in OI during ventilation (*p* = 0.004). Using Pediatric Acute Lung Injury Consensus Conference and pediatric acute respiratory distress syndrome thresholds, survival likelihood declined with increasing severity, with no survivors in the severe category (OI >16). Similar trends were observed using updated Pediatric Acute Lung Injury Consensus Conference‐2 criteria and acute respiratory distress syndrome severity classifications.

**Conclusions:**

Higher OI values and lower PF ratios were associated with mortality in this group of mechanically ventilated dogs, with both metrics demonstrating similar predictive accuracy. Results suggest species‐specific OI and PF thresholds are needed.

**Abbreviations**
ABG: arterial blood gas
ARDS: acute respiratory distress syndrome
AUC: area under the receiver operating characteristic curve
MAP: mean airway pressure
OI: oxygenation index
PALICC: Pediatric Acute Lung Injury Consensus Conference
PARDS: pediatric acute respiratory distress syndrome
PEEP: positive end‐expiratory pressure
PF: partial pressure of oxygen in arterial blood/fraction of inspired oxygen
PIP: peak inspiratory pressure

## 1 Introduction

In mechanically ventilated patients, the PaO_2_/FiO_2_ (PF) ratio, which calculates the PaO_2_ relative to the FiO_2_, is a widely recognized tool for assessing acute hypoxemic respiratory failure [1, 2, 3]. However, the PF ratio does not account for mean airway pressure (MAP), a key factor that can meaningfully affect oxygenation [1, 2, 3, 4, 5]. MAP is determined by peak inspiratory pressure (PIP), positive end‐expiratory pressure (PEEP), and the inspiratory‐to‐expiratory time ratio [6, 7]. It is influenced by reduced pulmonary parenchymal and chest wall compliance, increased airway resistance, and increased dead space ventilation, reflecting the mean alveolar pressure across both inspiration and expiration [2, 3, 4, 6, 7, 8].

An alternative metric, the oxygenation index (OI), incorporates MAP and is expressed as OI = (MAP × FiO_2_ × 100) / PaO_2_ [1, 2, 3, 4, 5, 7, 8, 9]. OI may provide a more comprehensive assessment of lung dysfunction and has been integrated into the diagnostic criteria for pediatric acute respiratory distress syndrome (PARDS) [4, 5]. In 2015, the Pediatric Acute Lung Injury Consensus Conference (PALICC‐1) defined OI‐based thresholds for PARDS, with mild PARDS classified as 4 < OI < 8, moderate as 8 < OI < 16, and severe as OI ≥16 [4]. The guidelines were further updated in 2023 (PALICC‐2), consolidating mild and moderate PARDS into an OI <16, while severe PARDS was defined by an OI ≥16 [5].

Studies in critically ill neonatal, pediatric, and adult human patients have demonstrated that OI is associated with both the duration of mechanical ventilation and mortality [1, 2, 3, 4, 5, 7, 8, 9, 10, 11, 12]. In veterinary medicine, the evaluation of OI has been limited to a single retrospective study, which found that OI at the time of initial ventilator stabilization correlated with successful weaning, with a lower OI associated with better outcomes [13]. However, the study did not assess the prognostic accuracy of OI or compare its performance directly with the PF ratio.

The objective of the current study was to evaluate OI values of survivor and nonsurvivor dogs mechanically ventilated for primary pulmonary parenchymal diseases. A secondary objective was to stratify patients into mild, moderate, and severe hypoxemia groups using established OI (PARDS) and PF (acute respiratory distress syndrome [ARDS]) ranges and attempt to correlate these with mortality risk. We hypothesized that higher OI values and lower PF ratios would be strongly associated with mortality, and OI would demonstrate superior discriminatory ability between survivors and nonsurvivors.

## 2 Materials and Methods

This retrospective, multi‐institutional, observational study included dogs that underwent mechanical ventilation between July 2020 and July 2024. Animals were excluded if they had incomplete data collection sheets, PaO_2_ measurements without simultaneous MAP or FiO_2_ values recorded (i.e., data within 1 h of arterial blood gas [ABG] analysis), and mechanically ventilated patients in which arterial samples could not be obtained.

Data extracted from medical records included MAP, FiO_2_, and PaO_2_ values. OI and PF ratios were calculated from simultaneously obtained ABGs and recorded FiO_2_ and MAP values using non‐temperature‐corrected values^1^^,^ 2 ^,^ 3 ^,^ 4. Patients were categorized into mild, moderate, or severe hypoxemia groups based on PALICC‐1 and PALICC‐2 thresholds for PARDS. PALICC‐1 defined mild PARDS as 4 ≤ OI < 8, moderate as 8 ≤ OI < 16, and severe as OI ≥17, while PALICC‐2 consolidated these into mild to moderate PARDS (OI <16) and severe PARDS (OI ≥16). Human ARDS thresholds were also applied using PF ratios (mild: <300, moderate: <200, severe: <100) [14, 15]. These classifications were assessed on Day 1, throughout the ventilation period, and on the final day.

Additional clinical data retrieved included signalment, body weight (kg), primary diagnosis, indication for mechanical ventilation, ABG sampling time, hospitalization length, duration of mechanical ventilation, and survival to discharge. Additional ventilation parameters included PIP (cm H_2_O), PEEP (cm H_2_O), inspiratory tidal volume (TVi; mL), patient respiratory rate, and set respiratory rate. Additional data from ABGs included PaO_2_ (mm Hg) and PaCO_2_ (mm Hg).

### 2.1 Statistical Analysis

Categorical variables are reported as counts and percentages. Continuous variables were evaluated visually and by skewness and kurtosis tests to determine whether they are normally or nonnormally distributed and are reported as mean (±SD) and median (range), respectively. Continuous variables in two independent samples, such as survivors and nonsurvivors, were compared with the two‐sample *t*‐test if the variable was normally distributed or with the Wilcoxon–Mann–Whitney test if the variable was nonnormally distributed. Simple logistic regression was performed to determine whether the median OI and PF, both on the first day and over the duration of mechanical ventilation, could predict the binary outcome of survival versus nonsurvival. The Liu method was applied to identify an empirical optimal mortality predictor cutoff point, and the corresponding area under the receiver operating characteristic curve (AUC), along with sensitivity and specificity, was calculated for the optimal cutoff point. A *p*‐value of <0.05 was considered significant for all tests. All statistical evaluations were performed using statistical software^5^.

## 3 Results

One hundred fifty‐three dogs required mechanical ventilation between July 2020 and July 2024. Seventy‐nine dogs met the inclusion criteria, while 74 were excluded for lack of arterial blood sampling (44 dogs; 28.7%); a nonprimary pulmonary parenchymal indication for mechanical ventilation (17; 11.1%); absence of simultaneous MAP or FiO_2_ recordings within a 1‐h period of arterial blood–gas analysis (8; 5.2%); and missing respiratory data (i.e., MAP, PaO_2_, or FiO_2_) (5; 3.3%). There were 619 simultaneously recorded PaO_2_, MAP, or FiO_2_ values.

Of the 79 dogs included, 27 (34.2%) were neutered females, four (5.1%) were intact females, 34 (43.0%) were neutered males, and 14 (17.7%) were intact males. The median weight was 16.0 kg (range: 2.3–69.9 kg). The median age was 6 years (range: 3 months to 14 years). Seventeen breeds were represented in the study sample, including 21 (26.6%) mixed‐breed dogs, which was the most common breed. Also included were 10 (12.7%) Bulldogs, eight (10.1%) French Bulldogs, seven (8.9%) Golden Retrievers, five (6.3%) Dachshunds, four (5.1%) Yorkshire Terriers, three (3.8%) Great Danes, and three (3.8%) German Shepherd Dogs. Eighteen (22.8%) other breeds, with one dog from each, comprised the remaining patients.

The most common indications for mechanical ventilation are displayed in Table 1. Thirty of 79 (38%) dogs survived to discharge, while 49 (62%) either died (34; 43%) or were euthanized (15; 19%). The median duration of mechanical ventilation was 2 days for both survivors and nonsurvivors (range: 1–6 days for survivors, 1–16 days for nonsurvivors; *p* < 0.05).

**TABLE 1:** Indications for mechanical ventilation in 79 dogs with primary pulmonary parenchymal disease.

Indication for mechanical ventilation	Frequency (*n* = 79)	Percentage of total population (%)	Survival, % (*n/N*)
Pneumonia	48	61.0	42 (20/48)
Acute respiratory distress syndrome	7	9.0	14 (1/7)
Cardiogenic pulmonary edema	6	7.5	50.0 (3/6)
Noncardiogenic pulmonary edema	4	5.0	50.0 (2/4)
Pulmonary contusions	3	4.0	66.6 (2/3)
Submersion injury	2	2.5	0.0 (0/2)
Other disease	9	11.0	40.0 (2/5)

Throughout mechanical ventilation, the median OI values were lower in survivors (median: 2.6; range: 1.2–11.2) compared with nonsurvivors (median: 6.6; range: 1.3–29.6) (*p* < 0.001) (Table 2). A critical threshold for survival was observed at an OI of 11.15, above which no patients survived. The median PF was significantly higher in survivors (median: 317.9; range: 86.6–541.7) than nonsurvivors (median: 177.9; range: 48.5–487.2) (*p* < 0.001) (Table 3). The median OI had an AUC of 0.73, with sensitivity and specificity of 65% and 80%, respectively, at an optimal cutoff point of 4.3. The median PF had an AUC of 0.72, with sensitivity and specificity of 70% and 73%, respectively, at an optimal cutoff point of 237.8.

**TABLE 2:** Comparison of median and maximum OI between surviving and nonsurviving dogs at different time points during mechanical ventilation, including the change (Δ) from Day 1 to the final day, in 79 dogs undergoing mechanical ventilation for primary pulmonary parenchymal diseases.

Median OI	Survivors (*n* = 30)	Nonsurvivors (*n* = 49)	*p*‐value
All days	2.6 (range: 1.2–11.2)	6.6 (range: 1.3–29.6)	<0.001
First day	3.2 (range: 1.1–14.6)	7.9 (range: 1.0–32.2)	<0.001
Last day	2.1 (range: 0.8–11.2)	6.9 (range: 1.6–36.2)	<0.001
Δ First and last day	−0.4 (range: −0.3 to 11.8)	0 (range: −21.2 to 29.2)	0.014

Abbreviation: OI, oxygenation index.

**TABLE 3:** Comparison of median and maximum PaO_2_/FiO_2_ ratios between surviving and nonsurviving dogs at different time points during mechanical ventilation, including the change (Δ) from Day 1 to the final day, in 79 dogs undergoing mechanical ventilation for primary pulmonary parenchymal diseases.

Median PF	Survivors (*n* = 30)	Nonsurvivors (*n* = 49)	*p*‐value
All days	317.9 (range: 86.6–541.7)	177.9 (range: 48.5–487.2)	<0.0001
First day	251.05 (range: 57.45–567.1)	123.8 (range: 34.1–520)	<0.006
Last day	324.825 (range: 86.55–588.4)	166.05 (range: 40.9–487.2)	<0.001
Δ First and last day	6.9 (range: −56.5 to 520.6)	0.00 (range: −356.3 to 337.1)	0.02

Univariate logistic regression found that the median OI during mechanical ventilation predicted mortality (*p* = 0.001), with each 1‐unit increase associated with 35% higher odds of death (odds ratio [OR]: 1.35; 95% confidence interval [CI]: 1.14–1.61). Similarly, the median PF predicted mortality (*p* = 0.001), with each 25‐unit increase associated with 23% higher odds of death (OR: 1.009; 95% CI: 1.004–1.013).

On the first day of ventilation, the median OI was lower in survivors (3.2; range: 1.1–14.6) compared with nonsurvivors (7.9; range: 1.0–32.2) (*p* < 0.001) (Table 2). Similarly, the median PF was higher in survivors (251.1; range: 57.5–567.1) than nonsurvivors (123.8; range: 34.1–520) (*p* < 0.001) (Table 3). The AUCs for OI and PF on Day 1 were both 0.69, with sensitivity and specificity, respectively, 84% and 52% (OI cutoff: 3.25) and 73% and 65% (PF cutoff: 183.85).

On the final day of mechanical ventilation, the median OI in survivors (2.1; range: 0.8–11.2) remained lower than in nonsurvivors (6.6; range: 1.6–36.2) (*p* < 0.001) (Table 2), while the median PF ratios in survivors (324.8; range: 86.6–588.4) were higher than in nonsurvivors (166.1; range: 40.9–487.2) (*p* < 0.001) (Table 3). The AUC for OI on the final day was 0.81 (sensitivity 80%, specificity 83%; cutoff: 2.98), while the AUC for PF was 0.78 (sensitivity 83%, specificity 73%; cutoff: 237.15).

The median change (Δ) in OI values from the start to the end of mechanical ventilation was different between survivors and nonsurvivors (survivors: median Δ: −0.4; range: −0.3 to 11.8; nonsurvivors: median Δ: 0.0; range: −21.2 to 29.2) (*p* = 0.014) (Table 2). The median Δ PF ratio was also different between survivors and nonsurvivors (survivors: median Δ: 6.9; range: −56.5 to 520.6; nonsurvivors: median Δ: 0.0; range: −356.3 to 337.1) (*p* = 0.02 (Table 2).

Survival decreased with increasing PARDS severity (Table 4). Based on PALICC‐1 definitions, the highest survival was seen in patients without PARDS (OI <4) and with mild PARDS (4 ≤ OI < 8), while survival decreased in the moderate PARDS category (8 ≤ OI < 16), with no survivors in the severe category (OI ≥16). Using the updated PALICC‐2 definitions, survival was limited to patients in the mild‐to‐moderate category (OI <16), with no survivors in the severe category (OI ≥16). Similarly, survival decreased with increasing ARDS severity (Table 5), with the highest survival observed in patients without ARDS, followed by those with mild ARDS. Survival further decreased in moderate ARDS, with a sharp decline in severe ARDS. Detailed survival and nonsurvival distributions across PARDS severity are presented in Table 5.

**TABLE 4:** Survival and nonsurvival based on PARDS severity using PALICC‐1 and PALICC‐2 definitions in 79 dogs undergoing mechanical ventilation for primary pulmonary parenchymal diseases.

PARDS severity	Survivors (%)	Nonsurvivors (%)
PALICC‐1 definitions		
No PARDS (OI <4)	56.4 (22/39)	43.6 (17/39)
Mild (4 ≤ OI < 8)	35.3 (6/17)	64.7 (11/17)
Moderate (8 ≤ OI < 16)	13.3 (2/15)	86.7 (13/15)
Severe (OI ≥16)	0 (0/8)	100 (8/8)
PALICC‐2 definitions		
Mild/moderate (OI <16)	42.3 (30/71)	57.7 (41/71)
Severe (OI ≥16)	0 (0/8)	100 (8/8)

*Note*: Percentages are calculated based on the total within each OI category.

Abbreviations: OI, oxygenation index; PALICC, Pediatric Acute Lung Injury Consensus Conference; PARDS, pediatric acute respiratory distress syndrome.

**TABLE 5:** Survival and nonsurvival based on ARDS severity using the Berlin definition in 79 dogs undergoing mechanical ventilation for primary pulmonary parenchymal diseases.

ARDS severity	Survivors (%)	Nonsurvivors (%)
Berlin definition		
No ARDS (PF ≥300)	56.5 (13/23)	30.4 (7/23)
Mild (200 ≤ PF < 300)	42.9 (9/21)	71.4 (15/21)
Moderate (100 ≤ PF < 200)	30.4 (7/23)	69.6 (16/23)
Severe (PF <100)	8.3 (1/12)	91.7 (11/12)

*Note*: Percentages are calculated based on the total within each PF category.

Abbreviations: ARDS, acute respiratory distress syndrome; PF, PaO2 / FiO2.

## 4 Discussion

The results of the current study support the hypothesis that higher OI values and lower PF ratios are strongly associated with mortality in mechanically ventilated dogs. Each 1‐unit increase in OI was associated with 35% higher odds of mortality (OR: 1.35; 95% CI: 1.14–1.61, *p* = 0.001), highlighting the potential role of OI in risk stratification and as a prognostic marker.

Throughout ventilation, survivors consistently showed lower OI values (median: 2.6 vs. 6.6 in nonsurvivors; *p* < 0.001) and higher PF ratios (median: 317.9 vs. 177.9; *p* < 0.001). Although OI demonstrated slightly greater predictive accuracy (AUC 0.73) compared with PF (AUC 0.72), their performances were comparable in this population. These findings contrast with pediatric and adult ARDS studies, in which OI has been identified as the stronger predictive metric [4, 8, 9, 10, 11, 12]. This disparity may reflect differences in ventilation strategies between human and veterinary medicine. In human medicine, the ARDSnet protocol uses a wide range of PEEP settings, including low and high titration protocols up to 24 cm H_2_O, which influence OI as MAP is affected by PEEP. For example, two patients with identical PF ratios (200) but differing MAP values (20 cm H_2_O vs. 10 cm H_2_O) yield OIs of 10.0 and 5.0, respectively, reflecting more severe lung pathology with higher MAP and OI. Veterinary practices often adapt ARDSnet strategies for the management of pulmonary parenchymal diseases, emphasizing low tidal volumes and plateau airway pressures <30 cm H_2_O. However, PEEP rarely exceeds 15 cm H_2_O due to concerns for hemodynamic instability. In the current study, PEEP ranged from 3 to 15 cm H_2_O (median: 6 cm H_2_O), comparable to a previous study reporting median OI values of 2.5 in survivors and 6.9 in nonsurvivors, with a median PEEP of 7 cm H_2_O (range: 2–14 cm H_2_O; *p* < 0.0001) [13]. The narrower PEEP range in veterinary patients may explain the similar predictive performance of OI and PF ratios in this population.

Lower OI thresholds were associated with increased mortality in the current study, highlighting the differences from the higher thresholds used in pediatric and adult human studies. For children with severe PARDS (OI ≥16), mortality rates range from 30% to 50% [4, 5, 16], demonstrating that this threshold retains meaningful risk stratification in that population. In contrast, survival in this study was highest in patients without OI consistent with PARDS (OI <4 [56.4% mortality]) and declined progressively with increasing OI, with no survivors that would have fit the severe category (OI ≥16 [100% mortality]). These results suggest that an OI cutoff of 16 may be too high for effective risk stratification in dogs. Even the moderate category (OI 8–16), with survival of only 13.3%, may lack sufficient granularity for guiding clinical decisions, as it primarily differentiates survivors from nonsurvivors.

A potential explanation for the lower OI threshold in the current study, beyond the ventilation strategies previously discussed, is the heterogeneity of the underlying diseases. Although many veterinary patients with primary parenchymal disease are managed using low tidal volume ventilation and ARDSnet criteria, 61% of patients in this study were ventilated for severe pneumonia, and only 9% had ARDS as the coded diagnosis. Mechanical ventilation is typically initiated after conventional oxygen therapy fails, when patients experience persistent, severe hypoxemia (PaO_2_ <60 mm Hg) or impending respiratory failure. Many of these patients likely had concurrent ARDS at this stage, but diagnosing ARDS definitively, especially retrospectively, can be challenging. Consequently, comparing OI thresholds in people with ARDS with this heterogeneous population of dogs may not be directly applicable, potentially explaining the lower OI values observed.

Species‐specific physiologic factors may also partly explain the lower OI threshold observed in our study. Quadrupeds like dogs generally have a larger alveolar surface area relative to body size than people, facilitating more efficient gas exchange [17]. As a result, baseline OI values are inherently lower in healthy quadrupeds than in healthy human lungs. Therefore, even with severe primary parenchymal disease, veterinary patients may exhibit proportionally lower OI values. This physiologic difference underscores the need for species‐specific OI thresholds to more accurately assess respiratory failure severity and guide therapeutic interventions. Similarly, higher PF thresholds were associated with increased mortality, with survival decreasing as PF ratios declined. There was only one survivor in the severe ARDS category, suggesting that a PF ratio of 100 may be too low to provide meaningful risk stratification in veterinary medicine. These findings emphasize the necessity of refining species‐specific thresholds for both OI and PF ratios to improve risk assessment and clinical outcomes in veterinary patients.

When comparing OI on Day 1, throughout ventilation, and on the final day, the AUC—and thus the predictive capacity—steadily rose, increasing from 0.69 on Day 1 to 0.81 on the final day. On Day 1, OI values in some survivors were as high as 23, indicating that early OI and PF ratio measurements should not be used solely to predict mortality. Instead, these metrics should guide ventilator adjustments and efforts to address reversible lung injury. Elevated OI and reduced PF ratios on Day 1 may reflect oxygenation impairment caused by reversible conditions (e.g., atelectasis and pulmonary edema), which can be improved with interventions such as increasing PEEP to recruit collapsed alveoli. Day 1 metrics represent an opportunity for therapeutic intervention rather than definitive prognostication. In contrast, persistently elevated OI or minimal reductions in ΔOI during ventilation were more reliable predictors of mortality. Survivors showed significant improvements in OI over time, indicating resolving lung injury, while nonsurvivors exhibited persistently high OI and minimal changes, reflecting severe, non‐resolving damage such as fibrosis or non‐recruitable lung regions. These findings align with studies in people, in which sustained poor oxygenation metrics are associated with increased mortality and weaning failure [4, 8, 12], emphasizing the importance of trending change over time rather than relying on one‐off measures.

On the final day of ventilation, OI demonstrated its highest predictive accuracy (AUC 0.81), with survivors exhibiting significantly lower values (median: 2.1). Low OI reflects sufficiently improved lung function and oxygenation necessary for successful weaning from mechanical ventilation. Notably, the current study found survival to discharge of 38%, which is higher than previously reported outcomes for dogs ventilated for primary pulmonary parenchymal disease, in which previous literature cites a survival to discharge rate of approximately 20% [18]. This improvement may reflect advancements in veterinary critical care, including the adoption of human‐based lung‐protective strategies such as lower tidal volumes, lower driving pressure, and fluid restriction [19, 20, 21, 22]. Additionally, the underlying disease process remains a dominant determinant of prognosis, as evidenced by one veterinary study reporting that 50% of dogs with aspiration pneumonia were successfully weaned from ventilation compared with only 8% of dogs with ARDS, highlighting the disease‐specific responses to treatment [23]. In the current study, aspiration pneumonia was the most common diagnosis, accounting for 61% of the population, with survival in this subgroup of 42%, while only 15% of dogs with ARDS survived. These findings suggest that while improvements in clinical practices may contribute to better outcomes, the nature of the underlying disease continues to play a critical role in survival and weaning success in mechanically ventilated dogs.

There are several limitations to this study. The multicenter design introduced variability in data acquisition, treatment approaches, and ventilator strategies across the institutions. Additionally, the retrospective nature of the study made it difficult to definitively diagnose ARDS, complicating the use of both ARDS and PARDS criteria in a heterogeneous population with pulmonary parenchymal diseases. Furthermore, euthanasia and financial constraints, common in veterinary medicine, may have influenced mortality outcomes independent of disease severity. Standardized data collection in a future prospective study could help address these limitations and provide clearer insights into the prognostic use of OI in mechanically ventilated dogs. Although OI showed slightly greater predictive accuracy, the PF ratio performed comparably and offers the advantage of being easier to calculate, potentially enhancing its clinical applicability. Greater improvement in OI during ventilation was seen in survivors compared with nonsurvivors, highlighting the importance of monitoring trends versus relying on single measurements. The findings also emphasize the need for species‐specific thresholds, as physiologic differences, heterogeneous etiology, and unique ventilation strategies in veterinary medicine result in lower OI and higher PF values compared with human patients. Future research should focus on refining these thresholds for veterinary patients and evaluating their role in guiding therapeutic interventions.

## Author Contributions

Tereza Stastny, Deborah Silverstein, and Jiwoong Her contributed to conceptualization, data analysis and interpretation, drafting of the manuscript, and revisions. Curtis Rheingold contributed to data analysis and manuscript drafting. Rebecka Hess and Dana Caldwell contributed to manuscript drafting and revisions. Nick Jordan contributed to manuscript drafting and revisions. Jeongmin Lee contributed to data curation, investigation, and writing review & editing.

## Ethics Statement

The authors confirm that the ethical policies of the journal, as noted on the journal's author guidelines page, have been adhered to. No ethical approval was required as this is a retrospective study.

## Conflicts of Interest

The authors declare no conflicts of interest.

## Endnotes

1 Prime+ Vet STAT Profile, NOVA Biomedical, Waltham, MA.

2 Stat Profile pHOx Ultra, NOVA Biomedical, Waltham, MA.

3 epoc Blood Analysis System, Siemens Healthineers, Issaquah, WA.

4 Siemens Blood Gas Analyzer RAPIDPoint 500e CLIA, Brooklyn, NY.

5 Stata 14.0 for Mac, Stata Corporation, College Station, TX.
